# Supplementary figures and images for: Risk Allele Frequency Analysis of Single-Nucleotide Polymorphisms for Vitamin D Concentrations in Different Ethnic Group
Source: Genes (Basel). 2021 Sep 28;12(10):1530. doi: 10.3390/genes12101530 (PMC8536051; doi:10.3390/genes12101530)

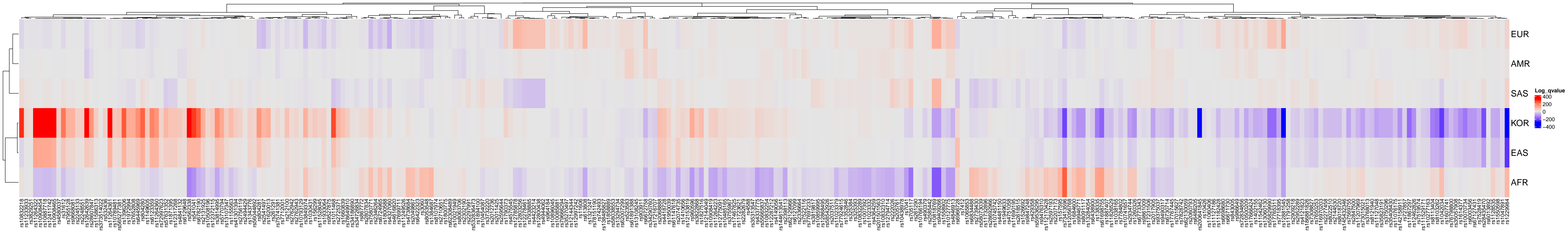

Supplement: Supplementary file 1 [file genes-12-01530-s001.zip › Figure S1.pdf]
